# The Circulation and Arsorption

**Published:** 1826-10-01

**Authors:** 


### MEDICO-CHIRURGICAL

### REVIEW.

VOL. IX.] Analytical Series. [No. 26.

" Nec tibi quid liceat sed quid fecisse decebit

" Occurrat, mentemque domat respectus honesti."

VOL. V.] OCTOBER 1, 1826. [No. 10.

[NEW SERIES.]

### I.

1. *Experimental Researches on the Influence exercised by Atmospheric Pressure upon the Progression of the Blood in the Veins, upon that Function called Absorption, and upon the Prevention and Cure of the Symptoms caused by the Bites of Rabid or Venomous Animals. (Dedicated by permission to His Majesty.) With an Appendix, containing the Original Reports of Baron Cuvier and of Professors Dumeril and Laennec, to the Royal Institute of France, and to the Royal Academy of Medicine of Paris, &c. &c.* By DAVID BARRY, M.D. Knight of the Order of the Tower and Sword, Member of the Royal College of Physicians in London, First Surgeon to the Portuguese Army, Surgeon to the Forces, &c. 8vo. pp. 174. Plates. London, 1826.

2. *The Syphonic Theory, or Brief Observations on the Circulation of the Blood, and on Respiration as connected therewith.* By EDWARD HOPLEY, Surgeon, R.N. 8vo. sewed, pp. 40. 1825.

DR. BARRY has done honour to himself and to his country, by a suite of ingenious experiments on the functions of circulation and absorption. The experiments on the *latter* have already led to most important practical conclusions in respect to the management of poisoned wounds, and will, we believe, transmit Dr. Barry's name to posterity as a philanthropist who shall have—"diminished the amount of human evils by increasing the stock of human knowledge."—It is with the subject of absorption, therefore, that we shall principally occupy our time and that of our readers in this article—not that we consider the *phenomena* of the circulation as explained by the perfect knowledge which we have of the *machinery* employed in that process—but because we fear that the process itself is still enveloped in as great mystery as ever. Harvey demonstrated, beyond all possibility of doubt, the *course* of the circulation—but not the *cause* of it. In making the heart the sole agent, we believe he erred. We think the vital and elastic powers of the arteries contribute to the blood's progression in those vessels—but a cloud hangs over the causes which move the blood from the extremities of the veins to their termination in the heart.—Various theories, or rather conjectures, have been formed from time to time, to account for the venous circulation—as the power of the heart, or vis à tergo, (which, indeed, is that of Harvey) the joint power of heart and arteries—some mysterious power in the capillary system—and lastly, a suction influence (the removal of atmospheric pressure) in the heart itself, or in some part of the machinery of respiration. This last power has been imagined, but never proved by experiment, till the time of Dr. Barry. He has, therefore, all the merit of a discoverer—provided the doctrine be true.

But before giving our readers a general outline of Dr. Barry's theory, we shall stop a moment on the subject of experimenting upon living animals. We need hardly say that we expressed ourselves in the strongest language which we could employ against the vulgar outcry ("not entirely unsupported," as Dr. Barry truly observes, "by some leading professional men") which was lately raised against every thing like inquiry, having for its basis *direct experiment upon living animals*. From the general silence that has reigned since that protest, among the ultra-Brahminical portion of the profession, we conclude that they are a little ashamed of their inconsistent philanthropy, and that we shall hear no more vociferations against the pretended cruelty of vivisections—a cruelty which has been practised by the wisest and most virtuous men of the ages in which they lived. The experiments of Harvey were honoured by the presence of his sovereign ("in jugulari vena interna denudata damæ vivæ (coram multis nobilibus, et rege serenissimo domino meo, assistentibus) per medium divisa, &c."—*De Circ. Sang.*) who placed an unlimited supply of animals at his disposal. The illustrious Bacon, too, in drawing a picture of what a university ought to be, puts the following words into the mouth of one of the Patres Domus Salomonis: "Habemus etiam septa et vivaria pro bestiis et avibus omnigenis, quibus, non tam propter novitatem et raritatem, *quam ad dissectiones et experimenta anatomica utimur*, ut ab iis, quid fieri possit circa corpus humanum lucem accipiamus, &c."—*Nova Atlantis.* Haller, also, who had at least as much real religion and true piety as some of those hyper-sensitive surgeons, "*qui nihil nisi homines secant*,"* gives the following as his opinion:—"dissecanda ergo animalia, verum minime sufficerit *cadavera* dissecuisse, *viva* incidisse necesse est."

"They," says Dr. Barry, "who inveigh most loudly against experiments upon living animals, and who affect an excess of sensibility, have never made any experiments themselves. They are contented with the exposition of what they, in their wisdom, suppose nature *ought* to do, instead of investigating what she actually does.

"Others talk of needless cruelty. If any useful knowledge is to be obtained by an experiment, none of the means necessary to arrive at this knowledge can be needless, and none else can be adopted without defeating the purpose aimed at; therefore, in useful experiments, there never is needless cruelty, or, in other words, unnecessary pain inflicted.

"When medical men are praised at public meetings, and their letters there read with applause, in which they profess the determination, neither to open the living book of animal nature themselves, nor permit it to be opened by the youth committed to their charge, our best feelings are allowed to take a very wrong direction. *There are those, however, who have had the candour and the honesty to assert in the face of this vulgar clamour, that we have as good a right to make animal life subservient to the increase of our useful knowledge, as of our bodily strength and amusements.* This is plain common sense, and must in the end prevail."

We, at least, did not betray our duty by keeping silent when hypocritical cant was usurping the place of true philanthropy. But to return from this digression to the immediate subject of Dr. Barry's Essay.

1. The Venous Circulation. Some vague and unauthenticated notion that the return of blood by the veins was in some way influenced by *suction*, may be traced as far back as Haller, who, with others, had noticed a marked coincidence between the respiratory movements of the thorax, in the warm-blooded mammalia, and the motion of their venous blood. But

* Harvey.

the mechanism by which Nature applies the agency of atmospheric pressure to the veins, and connects the expansion of the chest with the afflux of centripetal fluids to the heart, was never pointed out. Some of Dr. Barry's experiments, however, had been nearly approached, while others, as that upon the pericardium, had never been imagined by any man. But as we cannot do justice to Dr. Barry's reasonings or experiments within the limits which we propose to ourselves, we must refer to the work itself for a clear development of both the one and the other, contenting ourselves with a concise account of one or two experiments which, indeed, will give a pretty good insight into the scope and tendency of our author's theory.

*Exper.* 1. A horse, which had been condemned to death, was thrown on his right side, and the jugular vein laid bare and tied. An inch below the ligature a large-sized flexible catheter was introduced into the vein in a direction towards the heart, having a spiral glass-tube fitted into its outer end. When the horse was thrown, his breathing became almost entirely thoracic, and the rising and falling of the ribs could be distinctly counted. The respiration was almost audible. The catheter having been pushed towards the heart as far as it would go, a ligature which had been passed under the vein, was firmly knotted round both. The point of the spiral tube (on which the finger had been kept) was now immersed in a cup of solution of Prussian blue. The moment Dr. Barry removed his finger, the blue liquid rose through the spiral tube, and flowed rapidly towards the heart. The undissolved particles of blue were seen to pass up from the cup and round the tube during *inspiration*, and halt or return slowly towards the cup during *expiration*. Not a drop of blood was seen to enter the tube, but bubbles of air sometimes appeared upon the surface of the liquid in the cup during expiration.

Dr. Macann, who assisted in the experiment, was fully satisfied of the correctness of the foregoing statements.

"To vary the proofs of this wonderful coincidence between the movements of the blue liquid in the tube and the respiration of the animal, I withdrew the point C. from the liquid in the cup for a moment during *inspiration*, so as to admit one or two bubbles of air, and returned it again immediately. A space more or less extensive of the tube became thus transparent. Upon the next inspiration these bubbles were forced round the spiral with considerable velocity, and the whole tube again became uniformly blue by the ascent of more liquid from the cup. This part of the experiment, several times repeated, invariably afforded the same results." 13.

A considerable quantity of water and air had now been drawn into the circulation, and the animal gave strong signs of suffering. The experiment was, therefore, discontinued.

In the course of various repetitions of this experiment, Dr. Barry had occasion to remark, 1st. That, when the animal was standing, although the coloured liquid invariably rose in the tube, atmospheric pressure was never so distinctly marked as when he was prostrate. 2dly. The connexion between the motion of the liquid in the tube and the respiration could not be so satisfactorily observed while the horse was standing. 3d. When the respiration became hurried, from whatever cause, there was a frequent regurgitation of blood through the tube—a circumstance which never occurred except at the moment of expiration.

*Exper*. 2. Dr. Barry introduced into the thorax of a dog, near the median line, and on each side of the posterior extremity of the sternum, a metallic tube, pointed like a writing pen. The animal was placed on his back, and tubes were directed downwards and forwards parallel to the mediastinal pleuræ, suspending, in this position, the pericardium from the sternum. To the external extremity of each tube was attached a small caoutchouc bag filled with a composition of lard and wax, and pierced at its bottom by a small hole.

"As soon as the point of the tube had penetrated the pleura, I took a small flexible catheter, having at one end the barrel of a quill, in the side of which I had made a cut to act as a valve, opening readily from within outwards, and shutting in the contrary direction by its natural elasticity. The catheter thus armed, I passed into the hole in the caoutchouc bag, through the metal tube and into the chest. The little bag was attached to the margins of the wound by suture. This being done on both sides of the sternum, I next fitted to the outer end of each catheter which had been hitherto plugged, a spiral glass-tube, one end of which was already immersed in a coloured liquid. The communication being thus complete on both sides, the liquid rose rapidly through the spirals and flowed into the chest during inspiration, and remained stationary or fell during expiration. The movements of the liquid in the tubes were so regular, and so completely dependant upon the respiratory movements of the animal, that the one might be counted whilst observing the other. During inspiration I admitted into the glass-tube bubbles of air and small portions of the blue water alternately, so as to make the ascending column resemble a string of coloured beads, which played up and down through the spirals, particularly towards the latter part of the experiment, marking in a beautiful and striking manner the stages of the animal's respiration." 17.

Before the dog was destroyed, a stop-cock was fitted into his trachea, so as to command his respiration. When the stop-cock was shut, and the animal made powerful efforts to inspire, the blue liquid flowed upwards through the spirals with much greater force and rapidity than when the passage of the air through the windpipe was unobstructed.

*Exper.* 3. A similar communication still remained to be established with the bag of the pericardium, but, from some anatomical peculiarities in this animal, the experiments did not succeed. In the horse, however, Dr. Barry was enabled to pass a pointed tube along the surface of the xiphoid cartilage, through the lower margin of the diaphragm, and into the pericardium at its posterior and inferior angle, without penetrating the peritoneum. The tube was armed with a bag, as in the preceding experiment. Through this bag Dr. B. passed a flexible catheter into the tube, nearly to its point. Thus, when the pericardium was penetrated, the catheter could be pushed in immediately, and to any length, so as to prevent the heart from being wounded by beating against the point of the tube.

"In all the cases in which I succeeded in establishing a communication between the bag of the pericardium exclusively, and a coloured liquid, the fluid rose in the tube as rapidly as in the former experiments, and, in all but one, its motion upwards was governed by the animal's inspirations. In all, however, with the exception of this single case, although the liquid invariably halted or descended during expiration, there was an oscillation of the fluid upwards, which seemed independent of respiration, but could not be observed during inspiration, because then it was confounded with the general motion of the liquid upwards. This third movement was acknowledged by my friend Mr. Bennett, an anatomist and physiologist, as distinguished as he is modest." 21.

In this country, and at the Veterinary College, Dr. Barry repeated the first and third experiments, before Mr. Coleman, Mr. Sewell, and several eminent medical gentlemen, who all expressed their satisfaction at the entire success of the experiments—particularly that upon the pericardium. The operation was performed on a donkey. Some other experiments were made, but we have not space for them in this article. We shall now give Dr. Barry's conclusions in his own words.

"From what has been said, and from what has been observed in the experiments, the two following facts may be considered as proved:—

"*First*,—That the cavities of the great veins within the thorax, and all the thoracic cavities, draw towards them the fluids with which they are placed in direct communication.

"*Second*,—That this attraction, or suction, never takes place but during the expansion of the thorax, that is, during *inspiration*.

"From these facts, and from what we have seen in the last experiment, we may conclude—

"1st. That the blood which *runs contrary to its own gravity, arrives at the heart only during inspiration.*

"2dly. That the power which impels it at this moment through the veins, is atmospheric pressure.

"3dly. That as this power can be applied to the blood of the veins only at the time of inspiration, this blood must move with a velocity which is, to that of the blood moving through the arteries, as the time occupied by a whole respiration is to the time occupied by a single inspiration.

"4thly. As the blood passes through the greater veins during inspiration only, whilst it is incessantly traversing the arteries, it follows, that an accumulation must take place somewhere between these two orders of vessels, and that the quantity of this accumulation must be to the quantity which passes through the arteries during an entire act of respiration, as the time of one expiration is to that of a whole respiration.

"5thly. That, as it makes no difference with regard to the event, whether the accumulation which must be prepared for the expansion of the thorax, be made by two pulsations of the arteries or by ten, it follows, that the frequency of the pulse cannot be taken as the measure of the velocity of the blood returning to the heart, because it is the repetition of the inspirations which must regulate this velocity.

"6thly. That there are three quantities of blood; one passing through the arteries, one which is sucked up by each expansion of the thorax, and a third, which is collected during expiration between these two points. When therefore the respiration becomes hurried, this third quantity is diminished, whilst the other two are increased in proportion; but as the heart can admit only a certain quantity, the expanding cavities regurgitate the surplus during their collapse. Hence pathological phenomena, into which I shall not enter for the present.

"7thly. That the lymph and chyle must be sucked up towards the chest, through the direct communications which the vessels peculiar to these fluids have with the subclavian and other veins. The question of absorption, therefore, which has hitherto puzzled physiologists so much, may now be considered as decided, for it is clear that the open mouth of a vein, or of any other vessel, having the same kind of communication with the thoracic pumps, must absorb in direct proportion to the sucking power applied to it, and to the pressure exercised upon the matter to be absorbed.*

"If this last proposition be well founded, so ought to be the following corollary, *viz.*

"*That the application of a powerful cupping-glass to a recently-poisoned wound, would prevent the absorption of the poisonous matter.*

"8thly. It being now evident, from every thing that has been said, that the blood in the veins is placed under the influence of atmospheric pressure, it would be curious to trace the connexion which appears to

* See Experiment, No. 1, page 10.

exist between disease generally, intermittent fever for example, and the daily barometric variations.

"9thly. The preceding facts explain also why animal life cannot be maintained beyond a certain degree of atmospheric rarefaction, and why it must cease as soon as the pressure of the surrounding air ceases to be superior to the gravity of the column of blood. Birds are provided with a respiratory mechanism, which, in some measure, exempts them from this inconvenience.

"10thly. At the cardiac extremities of the great veins there exists, as we have shewn, a mechanism, which, when called into action by the expansion of the thorax, distends their cavities, and, consequently, causes the suction of the blood of the veins of the lesser, as well as of the greater, circulation. Now, as this mechanism can act only during inspiration, and as, from its construction, and its position, it must necessarily affect those portions of the auricles within the pericardium, called the sinus venosi, it follows that there can be no alternation of contraction between these parts of the auricles and the ventricles corresponding to the pulsa, because the sinus venosi must be in a state of progressive distension from the beginning to the end of *inspiration*." 39.

With this succinct view of Dr. Barry's experiments and conclusions relative to the venous circulation, we must leave the subject—acknowledging that those who wish to have a complete idea of this theory must peruse the original, with all its contributary elucidations. Our own impression is, that the motions of the chest, in inspiration, have very probably an influence on the current of the venous circulation, but the degree of influence does not appear to us of that magnitude which it does to the able and ingenious experimenter. The great drawback upon this and upon Dr. Carson's theory, is the circulation of the fœtus, where there can be no respiratory influence exerted, and yet where every thing goes on well. Dr. Barry has not yet been able to render any explanation of this formidable objection; but we understand he is preparing a work on the *whole of the powers* concerned in the movement of the blood in the different vessels, and till that appears we shall not enter into any examination of the theory of venous circulation propounded by this ingenious physician.

Before proceeding to the subject of absorption, however, we may just observe that Mr. Hopley, author of the little work entitled "the Syphonic Theory," at the head of this article, conjectured some years ago, "from the consideration of many particulars, that this operation (the flow of blood in the veins) must be brought about by the power of suction existing in the chest." As Mr. Hopley made no experiments to prove this—and as the conjecture is not original with him, we do not consider it necessary to say more on the subject at present.

II. Absorption. The knowledge of absorption may be traced in the history of poisoned wounds. How or when this baleful art originated is hidden from our researches—but it is evidently of the very earliest antiquity—and arrived at a much greater degree of *perfection* (if such a term can be allowed) in days of yore, than in the present scientific æra. The arrows of Hercules—the sufferings and death of Chiron, Nessus, &c. furnish so many direct proofs of the knowledge which man had acquired, in those remote ages, of the means of destroying his fellow creatures. The *manner* in which the mixture of the poison with the blood was effected, has not been recorded—at least till the times of Celsus and Galen. These physicians perceived that the veins were the fittest organs through which the matter from abroad could pass into the general system, and hence they recommended that ligatures should be applied above the poisoned wound, if on a limb. This view of external absorption prevailed for seventeen centuries, and doubtless the absorbing power was attributed to veins, arteries, and other vessels in common. In the middle of the eighteenth century, this power was first exclusively confined to the lymphatics. The authority of the Hunters overturned all the opinions of antiquity on this subject. But in recent times, Majendie has demonstrated the error of this exclusive doctrine, and the truth of the older one of venous absorption, as held by Celsus, Galen, Redi, Ruysch, &c. But Majendie did not satisfactorily shew the causes which induce or compel the matter deposited on a wounded surface to enter the cavities of the veins, and mix itself with the current of the circulation. Our author thinks that "the notions of a peculiar unintelligible vital power of discernment and appropriation, existing at the ends of the absorbing radicules, cannot even be alluded to."

We grant that it is rather difficult for the mind to endow the venous radicules into this elective capacity; but when we consider the peculiar and comprehensive powers of the lymphatics and lacteals, it will be as difficult to deny to *them* at least, such discriminating faculties.

M. Majendie proposed *imbibition* as the mode in which matter was transferred from the surface of a wound to the venous current:—thus, the matter, if solid, is first dissolved in the fluids of the wound, and when the coats of the vessels are soaked in the solution, that part of it which penetrates to their inside is washed off and carried forward by the current of the circulation. To this doctrine Dr. Barry objects the following difficulties.

"1. There must be a current flowing in the vein through the coats of which the imbibition takes place, else the imbibed matter cannot be washed off and carried forward.

"2. If the vein does contain a fluid, the imbibition or passive soaking of its coats may take place at least *as readily* from within outwards as in the opposite direction.

"3. The open mouth of a divided or wounded vein cannot become the subject of imbibition under any circumstances, and if the vessel be collapsed and empty imbibition will take place to no purpose, there being no current to carry forward the imbibed matter.

"4. In all wounds minute arterial and lymphatic branches must be divided and laid bare as well as veins, and as there can be no very great difference in the density of their coats, imbibition may take place through the sides of all, and consequently absorption if there be a current flowing through their tubes, but not otherwise," 81.

Nevertheless M. Majendie has unequivocally proved that matters *do* reach the circulation in some way or other, and, therefore, Dr. Barry observes that there must be some agent beyond mere *passive imbibition* to give this unvarying direction from without inwards, to a liquid applied to the surface.

At the present time, some, still hold that the lymphatics are the sole absorbents—some, that the sanguiferous veins alone perform this function—some that both are concerned in it. All know that absorption does take place—a fact that was known two thousand years ago.

In respect to the doctrine of absorption as applied to poisoned wounds, it would appear that little or nothing is said of these inflictions by Hippocrates, although he makes the first allusion to cupping-glasses, in the following words:—"*Cucurbitulæ, quæ eum in usum fabricatæ sunt, ut ex carne attrahant et avellant.*" In the Iliad, however, Machaon is made to suck the wound of Menelaus, which is the earliest record of a vacuum being applied to a wound, whether poisoned or supposed to be so. When the blood-vessels were pointed out as the channels for conveying the poison, ligature above the wound naturally suggested itself, and as the cucurbitulæ attracted towards them the contents of these vessels, their utility was rather confirmed than otherwise. Accordingly Celsus places the cucurbitulæ unequivocally at the head of all preventive and remedial agents, in cases of recently-poisoned wounds, as may be seen in the following passage. "*Utique autem, si rabiosus canis fuit, cucurbitula virus ejus extrahendum est; deinde, si locus neque nervosus, neque musculosus est, vulnus id adurendum est.*"

"For the bite of the viper, he recommends that a ligature should be immediately placed above the wound. '*Dein venenum extrahendum est.—Id cucurbitula optime facit.*' If, he adds, there should happen to be no cupping-instrument at hand, a circumstance which can scarcely be supposed as likely to occur, '*Homo adhibendus est, qui vulnus exsugat.*'

"These passages, and many others to be found in the same author, fully prove—

"1. That the *cucurbitulæ* were the chief, if not in his opinion, the only effectual means to be resorted to for the extraction of poison from wounds.

"2. That these instruments were so universally applied to this purpose at the time he wrote, that they were always to be found at hand.

"3. That direct suction by the mouth was next to cupping the best preventative, and that either of them was sufficient in cases of viper-bites; for in his directions upon this subject the cautery is not mentioned.

"After this the question of priority in the application of a vacuum to wounds inflicted by the bites of rabid and venomous animals, for the purpose of extracting the poison, can be entertained only by the antiquarian, and no man more modern than Celsus can be at all contemplated in the discussion of it." 86.

Strabo, Pliny, Galen, Plutarch, and many other ancients mention the Psylli, the Marsi, and the Ophigenes, as having the reputation of being born with the hereditary power of curing the bites of venomous serpents. The Psylli always sucked the wound, according to Celsus. It is well known that when Cato commanded an army in Africa, he hired and attached to his camp a certain number of these wound suckers; and it is recorded by Suetonius, that Augustus ordered the Psylli and Marsi to suck the wounds of Cleopatra, with the vain hope of restoring her to life, when she had just expired from the bite of a serpent. Redi follows Celsus to the letter—and Boerhaave, under the head ANTIDOTA, observes that poison may be removed from the body by various means—"in our days," says he, "*hodie per cucurbitulas magnas, validas, sæpe renovatas.*" He was among the last of the mechanical physiologists; for, about this time, the supporters of vital action and the absence of direct experiment upon living animals, produced a total change in the doctrines of external absorption. To the lymphatics this office was exclusively attributed, whilst the sanguiferous veins were refused all participation in this function. The consequent revolution which the treatment of poisoned wounds underwent was equally remarkable. The cupping-glass was laid aside as too mechanical. The lymphatics had taken up the poison by a peculiar *vital principle* inherent in them—their action must, therefore, be modified. Stimulants must be given to induce the exhalents to throw off the morbific matter—irritants must be applied to the wound, on the principle "*ubi stimulus ibi affluxus*"—the discharge was to be kept up by every possible means, whilst the vitality of the absorbents was to be destroyed by caustics. The knife and the hot iron was sometimes used;—but more frequently by the unlettered cow-leech than by the learned physician. From the days of Celsus to the present, Dr. Barry has not been able to meet with any record of a fair trial having been given to the application of the vacuum, either to the bite of the rabid dog, or venomous snake, although every author who has alluded to either of these subjects invariably mentions *cupping*, but merely as a secondary remedy. Orfila recommends *cupping* as a preventive measure in the bite of a mad dog, but does not mention it in the recent bite of the viper. Majendie and his followers do not appear to have founded any new mode of treatment on the doctrine of imbibition—and how far the injection of tepid water into the veins of animals labouring under hydrophobia may prove conducive to their recovery, is at present doubtful.

As a short resumé we may say that, from the times of Machaon to Celsus, we find but few and imperfect traces of any theory of absorption, while the treatment of poisoned wounds was hidden and disfigured by religious absurdities of the day—that from Celsus to Boerhaave, the blood-vessels were considered the channels of absorption, and to these vessels, therefore, the curative and preventive treatment of poisoned wounds was chiefly directed. The ideas that have prevailed since Boerhaave are sufficiently known—and now we appear to be coming back to the *practice* of Celsus, as well as to his doctrines.

"Yet, notwithstanding this, and many other modes of treatment equally inefficient and absurd, the plan of cure pursued by Celsus in cases of wounds inflicted by poisoned weapons, or by rabid or venomous animals, was beyond all comparison more successful than the mode of treatment adopted by the best physicians of the present day." 92.

A failure in preventing the ill effects of the bite of a venomous serpent, when suction had been continuously employed, was considered so remarkable, that Elianus recorded such an event in the case of a mountebank who was bitten in the arm by an aspis, and who died though he sucked the wound himself. It was recorded that the gums and palate of the mountebank mortified before his death. It is, therefore, probable that some ulceration existed in his mouth, which occasioned the failure.

In the second chapter of that part of Dr. Barry's work which treats of absorption, he enters into the questions whether this process can be strictly called a vital function—why it cannot take place *in vacuo*? Concluding that the immediate causes or circumstances indispensible to the accomplishment of absorption, are—*First*, A free communication between the matter to be absorbed and the thoracic cavities—*Secondly*, Atmospheric pressure, modified by the expansion of these cavities around one end of the communicating tubes, while the same pressure is free and undisturbed around the other end. With these data, and granting that both sanguiferous vessels and lymphatics are the organs of absorption, their communication with the thorax being exactly the same as that of the tube in the experiment alluded to, it was natural to presume that the absorption of any substance, as of a poison for instance, could not take place if the points of contact of the absorbing surface and of the matter to be absorbed, were placed under the influence of a vacuum. To put this to the proof our author procured several different kinds of poisons, the fatal activity of which had been well ascertained, such as prussic acid, strychnine, upas tieuté, arsenic, &c. With these and other poisons he experimented on rabbits and dogs, having generally two animals placed under exactly similar circumstances, except that the piston cupping-glass was applied to one, whilst the other was abandoned to his fate. The animal abandoned invariably perished within the periods stated. The animal to which the vacuum was applied never shewed the slightest symptom of poisoning, although the deleterious matter remained in contact with the wounded surface during the space of an hour, two hours, and even so long as five hours consecutively.

"When the poison was conveyed by means of a tube under the integuments to some distance from the opening by which it had been introduced, if the cupping-glass was applied to the sound skin, corresponding to the spot where the poison had been deposited (the wound being without the bounds of the vacuum,) not only was there no indication that any portion of the poison had been absorbed during the application of the glass, but even after it was taken off the animal continued for one or even two hours to carry imbedded in his cellular tissue a dose which would infallibly have destroyed him in a few minutes had the cupping-glass not been previously applied.

"In these cases, when I waited for the appearance of the tetanic convulsions, the reapplication of the glass immediately suspended them, and the removal of the poison through an incision in the integuments saved the animal.

"When I applied the cupping-glass over the opening made in the integuments for the purpose of introducing the tube, leaving the poison under the skin outside the bounds of the vacuum, no absorption took place during half or three-quarters of an hour, but as soon as the glass was removed absorption began.

"If, during the application of the glass, I made an incision between its edge and the point where the poison was placed under the integuments, absorption went on as if no vacuum were applied." 102.

A number of experiments made before some of the most eminent professors of Paris are next detailed, in proof of the foregoing statements, and for which we must refer to the work itself. Professor Laennec, who witnessed most of these experiments, drew up a report, in which, after recapitulating the principal ones, he comes to the following conclusions:—

"1st. Your committee is therefore of opinion that Dr. Barry's experiments (being the continuation of those by which he has already endeavoured to prove that the venous circulation is carried on principally under the influence of atmospheric pressure) establish, in the most incontestible manner, the influence of this agent on the circulation of the absorbent vessels, the proposition which the author sought to demonstrate.

"2ndly. That the knowledge of this important fact may be considered as a *real discovery*, notwithstanding the theoretical views and vague ideas entertained by some authors, and the empirical administration of suction to poisoned wounds, a practice more common with half-civilized people than more polished nations." 114.

The next suite of experiments are of a still more interesting nature, as exhibiting a still more direct application of this method of preventing poisoning by external absorption. Our author procured a considerable number of vipers from Grenoble and Fontainbleau, and had several dogs and rabbits bitten by these animals. To the bites of some he applied the cupping-glass—to the bites of others nothing; and, although the animals abandoned did not ultimately perish, the results obtained by the comparison were precisely analogous, as far as regarded the symptoms, to those observed in the preceding experiments—that is, the animals bitten by one, two, and sometimes three vipers, when the cupping-glass was applied for half an hour, suffered no symptom whatever of constitutional poisoning; whilst those that were left to nature were invariably attacked with convulsions, stupor, and the dogs by vomiting. Pigeons invariably perished from one bite of the viper, the fatal symptoms generally commencing before the end of the fifth minute; but, when the cupping-glass was applied immediately after the bite, they not only shewed no signs of having absorbed the venom while the glass remained on, but eventually escaped when the treatment to be noticed hereafter was adopted. We shall introduce a couple of experiments on the bite of the viper, which will give a good idea of the whole.

"On the 29th of September, 1825, in Baron Cuvier's anatomical laboratory, where, with his usual condescension, he was kind enough to permit me to avail myself of the talents and dexterity of M. Rousseau, Jun. one of his principal preparators, a large viper was applied* to the thigh of a half-grown weakly rabbit. The reptile bit twice: a minute drop of blood marked each puncture made by the fangs. One minute after the bites the piston-glass was applied upon the bitten part. M. Rousseau, who held his eye close to the glass whilst I worked the piston, observed a drop of transparent amber-coloured liquid issue from each of the punctures. This was followed by a considerable quantity of reddish serum, which rose into a thin froth, and in fifteen minutes nearly filled the glass with its large transparent bubbles. The vacuum was kept up for thirty-five minutes. When the rabbit was set at liberty he appeared to suffer no inconvenience: the little wounds presented nothing remarkable.

"One hour after this rabbit had been bitten the same viper was presented to the thigh of another, which he bit twice also, drawing blood, as before. The second rabbit was larger and much stronger than the first. A pale yellow spot was noticed almost immediately around each puncture made by the fangs. When the animal was set at liberty the bitten leg appeared slightly paralyzed. Ten minutes after the bite, the whole integuments of the bitten part appeared livid. Half an hour after, the lividity was intense, and had extended to the circumference of half a crown.

"The next day an open gangrenous ulcer occupied the whole of the livid circle, discharging a fetid sanies. The leg and thigh were swelled. Forty-eight hours after the bite, the ulcer was still open, but not so fetid. Seventy-two hours after the bites, the ulcer looking healthy, the limb reduced.

"During all this time, the rabbit first bitten never showed the slightest symptoms of either local or general poisoning. The second rabbit refused his usual food during the first thirty hours after he had been bitten, and looked dull." 125.

The second example which we shall introduce is selected on account of a precautionary measure of considerable importance which is there detailed.

"On the 24th October, two adult rabbits were bitten, each by three vipers, and by each viper three times. To one of these rabbits I applied the cupping-glass, which was left on thirty minutes. In this, as in No. 16, I observed a considerable quantity of serous fluid ooze

"* M. Rousseau applied the vipers by seizing them with a long forceps behind the posterior projecting angles of the head, and placing their nose in contact with the part intended to be bitten, they never failed to bite as often as we wished."

through the skin, and afterwards expand into thin froth with very large bubbles, filling the glass. I now dissected out the skin and cellular substance which had been included under the glass, applying the vacuum again for ten minutes; after which the wound was washed and the lips of it brought together by suture. The rabbit when set at liberty appeared to be in perfect health.

"The other rabbit had been left to his fate. On the 25th, at five in the afternoon, the cupped rabbit was as well as if nothing had happened to him: the wound in the thigh looking exactly as if it had never been touched by a viper's tooth, and inclining to heat.

"The rabbit that had been left to nature hung his ears, and looked very dull: the bitten thigh was much swelled, whilst a large gangrenous livid phlyctena, filled with a thin sanies, occupied the whole of the bitten part.

"On the 27th, the cupped rabbit in excellent health: the wound healing without any appearance of gangrene. The phlyctena in the other rabbit had degenerated into an extensive fetid ulcer. This animal after much suffering finally recovered." 129.

From these and many other experiments, whose results were so uniform, our author comes to the following conclusions, which we cannot abridge without detriment, and, therefore, shall give them in the words of Dr. Barry.

"*First.*—That neither sound nor wounded parts of the surface of a living animal can absorb when placed under a vacuum.

"*Second.*—That the application of the vacuum by means of a piston-cupping-glass placed over the points of contact of the absorbing surface, and the poison, which is in the act of being absorbed, arrests or mitigates the symptoms caused by the poison.*

"*Third.*—That the application of a cupping-glass for half an hour deprives the vessels of the part over which it had been applied of their absorbing faculty, during the hour or two immediately succeeding the removal of the glass.†

"*Fourth.*—That the pressure of the air forces into the vacuum, even through the skin, a portion of the matter introduced into the cellular tissue by injection; that is, if the skin of the animal be not too dense, as in the dog. (Exps. 16, 20.)

"From these facts we again arrive at the conclusions already established by the experiments detailed in Part I., viz.,—

1st. That *the taking up* of matter from the surface by the sanguiferous and lymphatic vessels, and the progression towards the heart of the contents of these vessels, are placed under the influence of atmospheric pressure, in all animals possessing thoracic cavities, and exercising over them the power of alternate contraction and dilatation around that point to which the centripetal current of their circulation is directed.

"2d. That, as the veins and lymphatics communicate with the tho-

"* Vide Exp. No 4." "† Vide Exp. No. 5."

racic cavities nearly in the same manner, the cardiac ends of both must be exempt from atmospheric pressure when the thorax is expanded, and therefore the pressure on the surface and extremities of these vessels being unresisted at this moment, except by gravitation, must not only press their contents upwards, but also force matter from abroad into their open mouths, or porous sides, when stript of their more dense coverings.

"3d. That as the height of the column of lymph exceeds that of the column of blood in the lower cava, by the distance from the lower point of the right auricle to the upper part of the subclavian vein in man, and as the course of the lymph is more tortuous and indirect (from passing through glands) than the course of the venous blood; it follows, that the velocity of the transport of matter from the surface to the centre, must be less in the lymphatic, than the sanguiferous veins, and that the comparative quantity transported by the two sets of vessels must be influenced by the circumstances already noted, and by the relative capacity of the vessels themselves. The difference in the specific gravities of blood and lymph should, perhaps, be also taken into calculation.

"4th. That as *imbibition*, *transudation*, *or passive soaking* of a part in a liquid may take place *in vacuo*, neither can be the agent which induces or compels matter deposited on the surface to penetrate into the cavities of the veins; for although the cupping-glass may arrest the current of the circulation in the smaller vessels during the period of its application, and even for some time after its removal, yet if imbibition could force the poison, which had been lying in the wound for hours, into their tubes, the washing of the part after taking off the glass would not save the animal from the effects of a substance which with the simple contact of the atmosphere would have killed him in a few minutes." 137.

In the succeeding chapter Dr. Barry makes some interesting remarks on the comparative absorbing powers of the different tissues—on morbid poisons—and on contagion and infection. He thinks it may be inferred that the absorbing powers of the tissues stand in proportion—*first*, to the pressure to which their veins are exposed—2d, to the freedom of communication with the thoracic cavities—3d, to the permeability of the mouths and coats of the veins—and, lastly, to the number of the veins. Accordingly we find that the membrane of the air-cells of the lungs absorbs with the greatest rapidity, because it unites in the most perfect degree the above conditions. Its veins are the most numerous—their communication with the central cavity of the thorax is the shortest and most direct—their coats are the most pervious—whilst their contents are forced forward by the whole pressure of the air rushing down the trachea during inspiration. Experiments illustrative of these observations will be found at pages 139-40 of Dr. Barry's work.

At the opposite extremity of the scale of absorbing tissues stand the osseous, fibro-cartilaginous, and epidermoid. In these there may be imbibition, but not absorption. Between the extremes of the scale may be ranged the subcutaneous cellular tissue—the mucous and the serous membranes. The conjunctiva absorbs freely, its vessels being numerous, and their coats thin.

"These experiments," observes Dr. B. "account for the communication of disease without contact. The infective matter of small-pox is more abundantly and more fatally taken into the system by breathing the atmosphere of the variolous, than by inoculation—the plague, by inhaling the effluvia of the pest-house. In short, whatever poison is capable of being suspended or dissolved in the air as a menstruum, must inevitably pass into the blood of those who respire this air thus infected. 'Qui cum non respirare non possunt, contagium miseri, evadere nequeunt.'"* 143.

Some poisons, as the vaccine virus, and most of those peculiar to brutes, are incapable of being dissolved in the atmosphere, at least in sufficient quantity to produce their usual effects. Others cannot be so concentrated as to affect the system through any other surface than that of the air-cells, such as the deleterious gases and effluvia. Some, again, are capable of infecting through all vascular tissues, though most fatally through the lungs, as the virus of small-pox and plague. In all cases, however, there is one common and indispensible condition, viz. "the contact of the poison with the surface through which it is to pass into the circulation." This circumstance, which we have always insisted upon, might check a little those noisy and minute distinctions which are eternally buzzing in our ears between contagion and infection—since they both come to the same thing in the end. The following passage contains sound reasoning.

"If one infected individual cannot furnish enough of virus to charge the atmosphere around him with the seeds of his disease, we know that a greater number can; and if the air be not disposed at one time to hold these germs suspended, we know that at other times it is so disposed. Therefore, whilst men have lungs constructed as these organs are at present—whilst the mucous surface of these lungs *are* exposed to the contact of every thing the atmosphere holds in solution—and whilst it is certain that the most fatal poisons may be thus deposited on the most rapidly-absorbing tissue of the whole frame, the healthy should be carefully and distantly separated from the infected; nor should they ever, under any circumstances, respire the air which the emanations from the latter may have poisoned.

"From what has been said on the subject of specific morbid poi-

* Galen.

sons, may be seen the incorrectness, nay, even the dangerous tendency of the distinction lately attempted to be established by some writers, between *contagion* and *infection*." 146.

Dr. Barry suggests (and we have no objection to the proposal) that, as the word *infect* and its derivatives clearly convey the idea of something noxious introduced into the system, without quibbling or ambiguity, they should, in sanitary logic, supersede the word contagion and its adjectives.

The sixth and last chapter of the work under review, is on the application of the foregoing principles and experiments to actual practice, in the treatment of poisoned wounds. The measures on which he comments are confined to such as are entirely physical and external. These are, 1mo. The ligature between the wound and the heart—2ndo. The cupping-glass or vacuum—3tio. Excision or scarification—4to. The actual cautery—5to. Protection from atmospheric pressure.

"1. In all cases of superficial poisoning, when the deleterious matter is simply deposited in the wound, the application of the cupping-glass over the point of contact will save the individual, provided it be made with the precautions to be noticed hereafter, and before a dose sufficient to cause death shall have been absorbed.

"2. In cases where the poison has been injected, as, for instance, by the hollow fang of a viper or rattle-snake, though the cupping-glass may have been applied, yet as the local action of the venom goes on in vacuo, the parts acted upon should be cut out after the venom has been concentrated and partly extracted by the cupping-glass, which should be immediately reapplied over the wound made by the knife, for the purpose of extracting the contents of the newly-divided vessels from a greater distance than could be done before the operation. After this the actual cautery may be administered, if thought necessary; but never under any circumstances before the second application of the cupping-glass, for this reason,—that when the mouths of the vessels are hermetically sealed by the hot iron, they can give out nothing to the vacuum.

"3. The poisoning that results from the bite of a mad dog, so far as regards the simple deposition of the deleterious matter in the wound, and the total absence of local action upon the wounded tissues, comes strictly under the first, or least complicated class of cases. But the tardiness with which the poison is absorbed, or if absorbed, with which it produces its peculiar effects, entitles it to be considered as a species *sui generis*.

"Fortunately this anomaly does not alter the preventative indications. These are purely physical, and as such must be ever unvaried. The first thing, then, to be done in treating the recent bite of a rabid dog is to apply a powerful cupping-glass over the wound. This measure supersedes at once the ligature, ablution, excision, &c. during the period of its application, and for a certain time after its removal.* 2. After the cupping-glass has been applied for an hour *at least*, the whole of the parts wounded or abraded by the bite should be freely dissected out. 3. The cupping-glass should then be reapplied immediately for the reasons already stated. 4. The wound should next be hermetically sealed by the actual cautery. 5. The part should be as little exposed to the contact of the air after the slough comes away, and as soon healed up, as possible." 150.

If the first application of the cupping-glass shall have so concentrated the poison, as that the excision of the part will remove it—or, if the second application shall have recalled such particles of it as may have been forced into the wounded vessels too far to be reached by the knife, but not beyond the limits of the influence of the vacuum, "the individual will be as secure against hydrophobia as if he had never been bitten." This, of course, is on the presumption that the nerves have nothing to do with the hydrophobic poison—a presumption which we believe to be correct. But still, as no experiments have been yet made on this curious and fearful poison, we think Dr. Barry is hardly authorised to make so confident an assertion as is contained in the foregoing passage. Dr. Barry thinks that the notion of the hydrophobic poison being absorbed in the usual manner, but lurking in the system for some weeks before it produces its peculiar effects, is contrary to all analogy. He believes it is with the rabid poison as with others—namely, that "the commencement of the symptoms is synchronous with the consummation of the absorption—and that their repetition is dependent upon its renewal." Certainly the idea of the removal of the poison in the wound is strengthened by the *general* occurrence of inflammatory symptoms in the part a few days previous to the explosion of the constitutional affection. But in very many instances there is no local warning of the dreadful scene which is at hand.

"Under the presumptive impression, then, that in hydrophobic, as well as in all other species of poisoning, the transport of the deleterious matter from the wound into the system, and the appearance of the symptoms peculiar to the poison, follow each other as cause and effect—as soon as the cicatrix begins to feel at all tender, or when there is sufficient evidence that the animal which inflicted the bite was rabid, we should immediately apply the cupping-glass, and proceed exactly as in the case of a recent bite; nor should the actual presence of hydrophobia deter us from this proceeding, any more than the presence of tetanic spasm in repeating the fourth experiment." 153.

"* Experiments 5—7."

It may be asked how it is that the cupping-glass should rank so low in the cure of poisoned wounds, seeing that its character has stood unimpeached since the days of Celsus? Dr. B. thinks that the answer is to be found in our ignorance of the mechanism of absorption, and our never having suspected that it was connected with atmospheric pressure. It is probable, too, that failure with the cupping-glass was attributable to improper interference with the poisoned wound—an interference which originally consisted in scarifications—actual or potential cautery—free exposure to the air. It is evident that the more exclusively the pressure is directed to the wounded surface, and the little vessels connected with it, the greater will be the probability of their contents being squeezed out into the vacuum. If scarifications surround the wound this object will not be so well attained. The objections to previous cauterization are so obvious that we need not dwell on them here. The only precautionary measures, previously to the application of the vacuum, are, according to our author, the ligature, not too tight, between the wound and the heart—simple ablution of the part—protection from contact with the air. "Even these measures are only to be used when a cupping-glass or suction by the mouth cannot be immediately commanded." We apprehend, that in these days of selfishness, few suckers of poisoned wounds will be found:—the cupping-glass, therefore, should be in the ready possession of every one—especially of the medical profession. Excision and the cautery can be of use only in proportion to their extent. If they reach beyond the poison they will certainly save, but not otherwise. The particles of poison which may have been already forced further than the boundary of the excised wound, will be sent to the heart with greater rapidity after the operation than they otherwise would have been (as was proved by Fontana) owing to the wider mouths of the vessels being now fully exposed to receive the atmosphere within their cavities. The following passage concludes the work.

"When the cupping-glass has been applied for an hour to the poisoned part, previously to removing it with the knife, the contents of all the vessels will have acquired a retrograde direction, and from not being permitted to flow freely into the vacuum, a perfect stasis of the fluids is established; hence the loss of the absorbing faculty of the cupped surface already noticed. (Experiments 5—7.)

"Thus by allowing the first cupping to precede the excision of the part, not only is there a greater quantity of the poison removed, but the danger of a more rapid absorption is avoided, whilst the certainty of extracting a still further portion, or, perhaps, the whole of what may have remained, constitutes an additional and important advantage to be obtained by the second cupping.*

"The advantage to be derived from the actual cautery, after the excision and second cupping, is also of a strictly physical nature. The burning of the little vessel hermetically closes its mouth, and renders its tube incompressible for a certain extent. Its absorbing powers, therefore, are suspended, because the pressure of the atmosphere can neither force any thing into it, nor compress it upon its own contents, so as to force them forward towards the heart." 159.

We have given such an ample analysis of Dr. Barry's work —especially that part of it which relates to absorption and poisoned wounds, that it would be quite superfluous to say any thing of its merits in conclusion. We confess that we are not converts to our ingenious author's theory of the circulation in the veins—and, consequently, in the absorbent vessels :—but, we nevertheless fully believe in the great utility and efficacy of the application of cupping-glasses to poisoned wounds. Whatever may be the power which causes the contents of veins and absorbents to go forward, under ordinary circumstances, towards the heart, there can be no doubt that the removal of atmospheric pressure from the mouths of these vessels when wounded, will cause a retrograde motion of the substances contained in them. The theory of absorption and venous circulation may be wrong, while the application of that very doctrine to practice may be very right. Under this impression, we beg to add our mite of approbation to that which the talented author has already so largely received abroad —recommending, in the strongest terms, the profession to procure for themselves the work, in order that they may be in complete possession of Dr. Barry's views—experimental—doctrinal—and practical.

---

* Experiments 22, 23.

